# Immunotherapy in non-small cell lung cancer: Update and new insights

**Published:** 2021-01-20

**Authors:** Xabier Mielgo-Rubio, Eider Azkona Uribelarrea, Laura Quintanta Cortés, María Sereno Moyano

**Affiliations:** ^1^Department of Medical Oncology, Hospital Universitario Fundación Alcorcón, Madrid, Spain; ^2^Department of Medical Oncology, Hospital Universitario Cruces, Bizkaia, Spain; ^3^Department of Medical Oncology, Hospital Don Benito-Villanueva, Badajoz, Spain; ^4^Department of Medical Oncology, Hospital Universitario Infanta Sofía, Alcobendas, Madrid, Spain

**Keywords:** immunotherapy, non-small cell lung cancer, lung cancer, immune checkpoint inhibitors, nivolumab, pembrolizumab, atezolizumab, durvalumab, PD1, CTLA-4

## Abstract

**Background:**

The treatment of non-small-cell lung carcinoma (NSCLC) has changed markedly in recent years as a result of two major treatment milestones: Targeted therapy and immunotherapy. Since 2015, immunotherapy has been changing the paradigm of NSCLC treatment in different settings and has contributed to improve the quality of life of these patients. The most widely used immunotherapy strategy in clinical practice is currently PD-1 and CTLA-4 immune checkpoint inhibition-based immunotherapy. Initial successful results came from an improvement in overall survival for pretreated patients, and immunotherapy subsequently moved to a first-line palliative setting as monotherapy, in combination with chemotherapy or as double-checkpoint inhibition. With regard to earlier stages, consolidation immunotherapy after chemoradiation has also changed the paradigm of unresectable NSCLC, with marked benefits in terms of disease-free and overall survival. During the last few years, efforts have focused on the introduction of immunotherapy in earlier stages as neoadjuvant treatment for potentially resectable tumors and in an adjuvant setting, with some very promising results.

**Aim:**

In this manuscript, we provide both an agile and thorough review of the role of immunotherapy in non-small cell lung cancer, a critical analysis of the most important studies, current indications, the role of biomarkers, new insights, and future challenges.

**Relevance for patients:**

Immunotherapy has revolutionized the treatment of non-small cell lung cancer patients reaching better survival outcomes in first and second palliative setting and in unresectable stage III tumors. Next year’s immunotherapy will also introduce in earlier stages. Through an extensive knowledge of the mechanisms of action and of immunotherapy-based studies, the best treatment alternative can be offered to patients, helping to improve their survival and cure rates.

## 1. Introduction

The treatment of non-small-cell lung carcinoma (NSCLC) has changed significantly in recent years, beginning with the discovery of oncogenic mutations as a molecular pathway responsible for some lung tumors, mainly not tobacco-related, for which anti-target therapies with excellent anti-tumor efficacy results have been developed. These therapies have helped to significantly increase survival and quality of life in those patients with tumors that carry these mutations, beginning with the discovery of EGFR back in 2004 [1,2], and have continued with the progressive discovery of new targets [3]. The other fundamental milestone that has helped the drastic and rapidly changing scenario of NSCLC, with a significant improvement in the overall survival (OS) of patients and an improvement in their quality of life, is immunotherapy [4], which is also changing the landscape of small cell lung carcinoma [5]. In 2013, the journal *Science* considered immunotherapy to be the scientific breakthrough of the year, and one of the disciplines in which it has undoubtedly advanced the most is NSCLC as a result of the use of PD 1 and CTLA-4 immune checkpoint inhibitors (ICIs) [6]. The first results showing an improvement in survival came from studies of NSCLC patients with advanced disease in progression to prior treatment with platinum-based chemotherapy, and this benefit was subsequently transferred to the first line of metastatic disease, both alone and in combination with chemotherapy (CT), as well as to locally advanced unresectable disease. In recent years, immunotherapy has also been rapidly introduced at earlier stages as neoadjuvant or adjuvant treatment for resectable and/or potentially resectable stages. Although generally well tolerated, immunotherapy can sometimes cause serious side effects that we must learn to manage [7]. Despite this, it nevertheless represents both an opportunity and a challenge, thus meaning that we must broaden our understanding of the molecular mechanisms that play a role in the antitumor immune response to better select those patients who will benefit most from it and to understand the mechanisms of resistance. Below, we will review the current situation of immunotherapy in each of the different scenarios in NSCLC and provide a critical analysis and vision of the future challenges in this field.

## 2. Cancer–immunity Cycle

Daniel S Chen was the first to describe the cancer–immunity cycle in his publication “*Oncology meets immunology: The cancer–immunity cycl*e.” This process begins with the release of tumor antigens by tumor cells, which can be recognized as foreign by the cells of the host immune system and ends with the destruction of these cells [8]. The immune response against cancer follows a “pseudomilitary” strategy, with seven differentiated steps: (1) Release and presentation of tumor neoantigens by tumor cells, with uptake of these antigens by antigen-presenting dendritic cells, which process them and reduce them to peptides. These peptides bind to the major histocompatibility complex (MHC); (2) recruitment of T lymphocytes at the peripheral lymphoid organs and presentation of peptides bound to MHC-I and MHC-II to T cells, with subsequent recognition of peptides bound to MHC-II by receptors on the CD4+ T lymphocytes; (3) training: priming; and activation of effector T cells to respond to the tumor antigens presented; and (4) attack with displacement of activated T cells to the region containing the tumor. After the specific activation of T cells in peripheral lymphoid organs, they need to be directed to the tumor through the endothelium and infiltrate the stromal tissue within the tumor, which requires certain phenotypic characteristics in the T cell, such as the expression of chemokine receptors or the expression of cell-adhesion molecules in the vascular endothelium that would allow the endothelial barrier to be overcome and the tumor to be invaded; (5) tumor infiltration; (6) recognition of tumor cells by cytotoxic T lymphocytes (T cell receptors need to come into contact with the MHC peptides on the surface of the tumor cell where, in the case of CD8 lymphocytes, they will release the granules containing cytolytic substances, such as perforin and granzyme, into tumor cells to destroy them); and (7) final destruction of tumor cells and release of new tumor neoantigens. Although it is possible to act on each therapeutic level during these phases, the strategies that have reached the clinic to date and have yielded initially successful results are monoclonal antibodies that block immune checkpoints CTLA-4 and PD-1, which control activation of the immune response at peripheral lymphoid organs and tumor level, respectively [9]. By blocking these immune checkpoints, the negative autoregulatory signal that blocks activation of the immune response is avoided, ultimately producing a stronger immune response with a greater number of active T lymphocytes ready to attack tumor cells. Although the greatest successes to date have been achieved with monoclonal antibodies that target these sites, other strategies that focus on other phases of the activation of the immune response, and combinations of different strategies, are under development [10,11]. Nivolumab, pembrolizumab, and sintilimab are anti-PD1 monoclonal antibodies, and atezolizumab and durvalumab are anti-PD-L1 monoclonal antibodies. Ipilimumab and tremelimumab are anti-CTLA-4 antibodies.

## 3. Immunotherapy in a Neoadjuvant Setting

The evidence for immunotherapy in early stages is limited. Major pathological response (MPR) is defined as the presence of ≤10% tumor cells in the resected specimen and has been adopted as a surrogate endpoint in neoadjuvant studies, where it has been seen to be predictive of higher OS [12]. One of the first studies was that of Forde *et al*. [13] who evaluated two cycles of nivolumab followed by surgery in stages I-IIIA. In this study, 20 of the 21 patients underwent surgery, with an MPR of 45% and a pathological complete response (pCR) of 10%. The response was correlated with TMB but not with PD-L1. Peripheral blood analysis identified tumor-specific T cells, which diminished over time, leaving a detectable percentage, which may reflect the possibility of long-lasting immunity. The *LCMC-3* study [14] analyzed two doses of neoadjuvant atezolizumab. In the analysis of 101 of the 180 planned patients, 90 underwent surgery. The MPR was 18%, with six pCR. A PD-L1 expression of ≥50% was correlated with response, but not TMB. The most promising results have been obtained in the phase II *NADIM* trial [15]. This trial evaluated three cycles of nivolumab combined with carboplatin + paclitaxel (C/P), followed by surgery and nivolumab for 1 year. With 46 patients included, 41 underwent surgery, and an MPR of 83% was observed, with 71% pCR. The phase II *NEOSTAR* study in stages I-IIIA compared neoadjuvant nivolumab as monotherapy or in combination with ipilimumab [16]. The MPR was 29% (10% with nivolumab and 43% with the combination). A total of 38% and 10% of patients, respectively, achieved pCR. A greater response was seen in those with greater PD-L1 expression. In patients who received the combination, the percentage of viable tumors was lower and there was a higher density of TILs. The results of a phase II trial in smokers with NSCLC stages IB-IIIA, who received four cycles of atezolizumab combined with nab-paclitaxel and carboplatin before surgery, have been published recently [17]. Of the 30 evaluable patients, 97% underwent surgery (87% R0), with an MPR of 57% and pCR of 33%, irrespective of PD-L1 expression. A phase Ib study in resectable stages IA-IIIB evaluated the administration of two cycles of sintilimab (anti-PD-1) before surgery [18]. The MPR was 40.5%, with a pCR of 16.2% in primary tumors and 8.1% in lymph nodes (a greater response was observed in squamous cells than in adenocarcinomas; MPR: 48% vs. 0%). The results of the phase II AFT-16 study, which evaluated four cycles of neoadjuvant atezolizumab followed by C/P concomitant to RT (60 Gys), followed by two cycles of consolidation with C/P and, subsequently, atezolizumab for up to 1 year, in 64 stage III patients, were presented at the ASCO 2020 congress. A response rate (RR) of 82% was achieved in PD-L1-negative patients and 90.9% in their PD-L1-positive counterparts [19].

These studies have shown a lack of concordance between the response according to RECIST criteria and the pathological response, with the former being lower in all cases. This is thought to be secondary to T-cell infiltration. Neoadjuvant immunotherapy has also been shown not to increase the number of surgical complications and to be well tolerated, with no delays in surgery or treatment withdrawals.

The spectacular results observed with immunotherapy combined with neoadjuvant CT, with an MPR of 57-80% (compared to 19% observed with CT), have promoted the initiation of numerous phase III studies. Several of these studies are currently ongoing (Table 1).

**Table 1:** Ongoing studies evaluating neoadjuvant immunotherapy

Trial	Phase	Stage	Trial setup
CheckMate 816 (NCT02998528)	3	IB-IIIA	CT vs. CT+Nivolumab
Impower 030 (NCT03456063)	3	IB-IIIA	CT+Atezolizumab vs. CT+Placebo
Keynote 671 (NCT03425643)	3	II-IIIB	CT+Pembrolizumab vs. CT
Aegean Trial NCT03800134	3	II-IIIB	CT+Durvalumab vs. CT+placebo
NEOSTAR NCT03158129	2	IA-IIIA	N vs. N+I vs. N+CT vs. I+N + CT
NADIM II NCT03838159	2	IIIA	N+CT vs. CT
NEOMUN NCT 03197467	2	II-IIIA	Pembrolizumab
NCT 03237377	2	IIIA	Durvalumab±Tremelimumab+RT

I: Ipilimumab, N: Nivolumab, CT: Chemotherapy, RT: Radiotherap.

The addition of immune enhancers is also being studied in the NEOCOAST (oleclumab, danvartisen, monalizumab), CANOPU N (canakinumab), and SHR1210 (apatinib) studies.

## 4. Immunotherapy in an Adjuvant Setting

Five-year OS rates in early stages vary from 50% in stage IA to 20% in stage IIIA with a still high relapse rate. The use of adjuvant platinum doublet-based CT has shown an improvement of 4–5% in OS at the expense of more than 50% grade ≥3 adverse events [20]. Therefore, there is a need to improve the results with the aim of generating prolonged antitumor responses, eradicating microscopic residual disease, and reducing local distal recurrences. As such, there is an interest in evaluating the role of immunotherapy in adjuvant NSCLC as it has previously demonstrated benefit in more advanced stages [21,22] and in the adjuvant treatment of other types of tumors [23]. Unfortunately, despite the significant progress made in the treatment of advanced NSCLC, the management of resected early disease has not witnessed any similar improvement. Despite this, a multitude of ongoing clinical trials may provide us with answers in the coming years. According to preclinical studies, although an immunosuppressive tumor microenvironment is already present from the very earliest stages [24], the results available from vaccine studies have not shown the expected outcomes [25]. Several phase III studies with immunotherapy, generally with ICIs, are currently underway to try to resolve this issue. Some of these are studying the value of immunotherapy after surgery and conventional adjuvant CT. Others, which are perhaps of more interest, are comparing CT treatment after surgery (control arm) with sequential CT-immunotherapy and with another comparator arm with concurrent CT-immunotherapy followed by maintenance immunotherapy for up to 1 year. This strategy is based on the rationale that the combination leads to an increase in tumor-specific immunogenic peptides and a potential increase in the immune response [26]. The primary objectives in most of these studies are OS and, in some cases, disease-free survival (DFS), thus leading to laborious studies that may only give us results once the therapy has become obsolete in this setting [13,27]. As such, it would be reasonable to search for surrogate markers to facilitate the conduct of these studies, as is the case in the neoadjuvant setting [13].

We have conflicting data from the predictive biomarkers of response in terms of both the level of PDL-1 expression [28-30] and *tumor mutational burden* (TMB) [31,32]. Liquid biopsy looks to be a promising tool. Circulating tumor DNA (ctDNA) has been found to be present in 50-95% of patients with resected stage I-III tumors; therefore, this could provide us with information in non-invasive monitoring of response to treatment and follow-up to recurrence [33]. In this regard, we should highlight the *ALCHEMIST* trial, which is currently recruiting, contains multiple arms, and involves genetic studies in stage IB-IIIA patients who have undergone, or will undergo, surgery, as it may help us in the ongoing search for biomarkers [34]. Similarly, the Spanish Lung Cancer Group (SLCG-GECP) will shortly launch an adjuvant study with immunotherapy and CT (Table 2).

**Table 2:** Ongoing studies evaluating adjuvant immunotherapy

Trial	N	Estimated completion date	NCT number
Adjuvant nivolumab in resected lung cancers – a randomized phase III study of nivolumab after surgical resection and adjuvant chemotherapy in treating patients with stage IB-IIIA non-small cell lung cancer (ANVIL)	903	July 2024	NCT02595944
Study of pembrolizumab (MK-3475) versus placebo for participants with non-small cell lung cancer after resection and completion of standard adjuvant therapy (PEARLS)	1080	February 2024	NCT02504372
Double blind placebo-controlled study of adjuvant MEDI4736 in completely resected NSCLC (BR.31)	1360	Enero 2024	NCT02273375
Study to assess safety and efficacy of atezolizumab (MPDL3280A) compared with best supportive care following chemotherapy in patients with lung cancer (IMpower010)	1280	December 2027	NCT02486718
Testing the Addition of a Type of Drug Called Immunotherapy to the Usual Chemotherapy Treatment for Non-small Cell Lung Cancer, ALCHEMIST Chemo-IO Study	1263	December 2024	NCT04267848
Genetic Testing in Screening Patients With Stage IB-IIIA Non-small Cell Lung Cancer That Has Been or Will Be Removed by Surgery (The ALCHEMIST Screening Trial)	8300	September 2021	NCT02194738

## 5. Immunotherapy in Inoperable Stage III NSCLC

Up until 2017, the definitive treatment in patients with unresectable tumors was definitive chemoradiotherapy (CTRT) based on the platinum doublet, irrespective of the histological subtype, and/or molecular characteristics. Moreover, no improvements had been seen after attempts to use induction and/or consolidation CT, biologics, antiangiogenics or vaccines.

ICIs have changed the landscape of unresectable stage III NSCLC with the addition of consolidation durvalumab after CTRT in an unselected population [35,36]. Updated data from the *PACIFIC* study have shown 36-month OS rates of 57% in the durvalumab arm and 43.5% in the placebo arm (HR: 0.68) [37]. However, when compared to the concurrent CTRT arm in the RTOG 9410 trial, the 36-month OS rate is only 28% [38]. After 24 months of follow-up, the objective DFS is 17.2 versus 5.6 months in the placebo arm (HR: 0.51). The median time to death or development of distal metastases is 28.3 versus 16.2 months in the placebo group (HR: 0.53), and a lower incidence of brain metastases is observed in the durvalumab group (6.3% vs. 11.8%). The objective response rates show a greater response in the immunotherapy arm (30% vs. 17.8%). Some data from patients treated in clinical practice confirm these results, which can be enhanced by adding locoregional treatments in the case of recurrence [39]. The PD-L1 expression level was not required for inclusion in the trial since, when the study was designed, no immunotherapy biomarker had been established and this value was only available for 63% of the patients included (22% with a level ≥25.0% and 41.0% <25.0%) [35]. A post-hoc analysis revealed that patients with PD-L1 expression ≥25% obtained a greater benefit in terms of DFS (HR: 0.41) and OS (HR: 0.50). The European Medicines Agency (EMA) also requested an unplanned post-hoc analysis based on PD-L1 expression levels in tumor cells in the initial biopsy, with a benefit being observed in terms of DFS irrespective of PD-L1 expression but no impact on OS in the specific PD-L1-negative subgroup (PD-L1 <1%). Based on these findings, approval was limited to the subgroup with PD-L1 ≥1%. Taking into account that it also stipulated a cut-off point for PD-L1 different to that established initially in the study (1% vs. 25%), and that this is a heterogeneous and dynamic biomarker that can therefore be influenced by CT and radiotherapy (RT) treatments, this was a controversial decision. As such, the use of durvalumab in patients with PD-L1 expression <1% is a subject of debate among the scientific community, with approval for the non-selected population in the United States and other countries, but with restrictions in Europe [40,41].

Another possible biomarker for which more robust studies are required is the mutation in STK11 that appears to confer a worse prognosis and inferior response to immunotherapy and may also affect patients treated with consolidation durvalumab [42].

Several studies have hypothesized the benefit of multimodal treatment. Indeed, preclinical data show an increase in PD-L1 expression after tumor irradiation and improvement in tumors receiving RT and immunotherapy [43-45]. Furthermore, tumor responses in areas not directly irradiated have been observed (abscopal effect) [46]. Although the *PACIFIC2* trial (NCT03519971) sought to address this assumption, it will nevertheless leave us with unsolved doubts given that it compares CTRT and consolidation durvalumab immunotherapy versus the control arm with CTRT and placebo, a treatment that is no longer standard [47]. However, recruitment for another study (NCT04092283) that should be able to answer this question has begun. In contrast, several ongoing studies are attempting to assess the hypothesis of whether RT and immunotherapy, with no CT, will be safe and effective in patients selected using biomarkers, as is the case in NSCLC stage IV (NCT03523702) [48].

Retrospective data from a limited number of patients, after a liquid biopsy study, appear to show a clonal expansion of T cells after CRT, and this may influence the immune response after PD-L1 blockade. Clonal expansion of regulatory T lymphocyte populations is associated with an increased likelihood of recurrence. The characterization of T-cell receptor (TCR) clones, as well as residual disease after treatment and the study of T-cell subpopulations, is currently underway [49].

Other ongoing trials, mainly involving anti-PD-1, anti-PD-L1, and anti-CLA4, are attempting to address issues such as the benefit of immunotherapy after sequential CTRT (DETERRED, NCT02525757 [50]; *PACIFIC-6* NCT03693300), the use of drugs at different doses and dosage regimens (*PACIFIC-5*, NCT03706690), induction with immunotherapy to continue with definitive CTRT (NCT03102242 with atezolizumab and NCT03285321 with nivolumab versus nivolumab and ipilimumab), as well as the benefit of combining immunotherapy with other agents (COAST, NCT03822351). Similarly, the optimal duration of immunotherapy needs to be defined as only 47% of patients completed durvalumab treatment in the *PACIFIC* trial [35]. It is also interesting to consider whether surgical rescue is possible after finishing the treatments and, if so, what would be the optimal moment to consider it. Another unanswered question is what would be the most suitable CT and RT scheme, as well as its duration. Assuming low statistical power, a retrospective analysis of 92 patients with unresectable stage III NSCLC treated with CTRT and subsequent pembrolizumab, which shows greater benefit of immunotherapy in stage IIIA and in those who receive a greater number of cycles of pembrolizumab, should be noted [51].

Another issue for debate and study is the safety and toxicity of the combination of RT and immunotherapy, specifically as regards pneumonitis [52]. In the *PACIFIC* study, for example, although rates of pneumonitis of any grade were higher in the durvalumab cohort (33.9% vs. 24.8%), there were no differences in the rates of grade 3-4 pneumonitis (3.4% vs. 2.6%) [35]. A recent study has found a statistically significant increase in radiation pneumonitis in patients treated with nivolumab who had previously received chest RT [53].

In conclusion, although we have witnessed a change in the treatment paradigm for unresectable stage III NSCLC, many questions remain unanswered.

## 6. Immunotherapy in Extensive NSCLC

### 6.1. ICI in untreated NSCLC

The relevant role of immunotherapy (anti-PD1/anti-PD-L1) in the treatment of patients with NSCLC and advanced disease has been clearly demonstrated in several randomized phase III studies published in recent years. Both immunotherapy as a single treatment, as well as the combination of CT and immunotherapy, or even double immunotherapy, have a defined therapeutic location, supported by favorable clinical trial results, as the first line of treatment for patients with advanced NSCLC (Table 3).

**Table 3:** Key studies in first-line NSCLC

Phase III study	Population	Treatment groups	HR, overall survival (m)
KEYNOTE-024	Untreated advanced NSCLC (no EGFR or ALK mutation) PD-L1 TPS _50%	Pembrolizumab versus Platinum-based chemotherapy	HR, 0.63 mOS, 30.0 months versus 14.2 months
KEYNOTE-042	Untreated locally advanced or metastatic NSCLC PD-L1 TPS_1%	Pembrolizumab versus Platinum-based chemotherapy	HR, 0.81 mOS, 16.7 months versus 12.1 months
KEYNOTE-189	Metastatic nonsquamous NSCLC (no EGFR or ALK mutation	Carboplatin/cisplatin pemetrexed pembrolizumab Carboplatin/cisplatin pemetrexed	HR, 0.49, 12-m OS, 69.2% versus 49.4%
KEYNOTE-407	Metastatic squamous NSCLC	Carboplatin/paclitaxel or nab-paclitaxel pembrolizumab Carboplatin/paclitaxel or nab-paclitaxel	HR, 0.64 mOS, 15.9 months versus 11.3 months
Checkmate 026	Stage IV or recurrent NSCLC PD-L1_1%	Nivolumab Platinum-based Chemotherapy	HR, 1.02 mOS, 14.4 months versus 13.2 months
Checkmate 227	Stage IV or recurrent NSCLC PD-L1 _1%	Nivolumab-ipilimumab versus Platinum-based chemotherapy	HR, 0.79 mOS, 17.1 months versus 14.9 months
IMpower 150	Metastatic nonsquamous NSCLC	Atezolizumab carboplatin/paclitaxel bevacizumab versus carboplatin/paclitaxel bevacizumab	HR, 0.78 mOS, 19.2 months versus 14.7 months
IMpower 110	Metastatic NSCLC (TC≥50% or IC≥10% stratified subgroup)	Atezolizumab versus cis/carboplatin pemetrexed (nonsquamous) or cis/carboplatin gemcitabine (squamous)	HR, 0,59 mOS 20.2 months versus 13.1 months

#### 6.1.1. Immunotherapy in monotherapy

To date, three positive studies have demonstrated an improvement in the efficacy of immunotherapy as monotherapy against CT, but in a profile of patients with high PD-L1 expression in two cases and with at least 1% PD-L1 expression in the other. The *KeyNote 024* study was the first to demonstrate the benefit of pembrolizumab at a fixed dose of 200 mg every 3 weeks versus platinum-based CT, in a population selected using PD-L1 expression in tumor cells (patients who presented PD-L1 expression >50% were included), excluding patients with activating mutations in EGFR or translocations in ALK, rather than by histology (adenocarcinoma and squamous carcinoma). Pembrolizumab administration was associated with a higher RR (44.8% vs. 27.8%), greater progression-free survival (PFS) (10.3 vs. 6.7 months), and a higher OS (30 vs. 14.2 months, HR: 0.63) [26,54]. The other study that demonstrated increased efficacy of monotherapy in high PD-L1 expressors was *IMpower 110*, which demonstrated a benefit of atezolizumab at a fixed dose of 1200 mg every 3 weeks versus platinum-based CT in patients with advanced NSCLC. This study included patients with PD-L1 expression ≥1 and one of the stratification factors was PD-L1 expression (TC3 or IC3; TC2/3 or IC2/3; TC1/2/3 or IC1/2/3). In the interim OS analysis with a median follow-up of 15.7 months, atezolizumab was superior to CT with an increase in OS of 7.1 months (HR: 0.595; *P*=0.0106) [55]. In addition to the role of anti-PD-1s as first line and in high expressing patients (PD-L1 >50%), the *KeyNote042* study explored the effect of fixed-dose pembrolizumab 200 mg every 3 weeks in a less restrictive population that included treatment-naïve patients with metastatic disease and PD-L1 expression ≥1%. Patients were again randomized to receive pembrolizumab versus platinum-based CT. The primary objective of the study was OS sequentially tested in different population subgroups based on PD-L1 expression, with a different magnitude of benefit in OS in all subgroups analyzed: PD-L1 >50% 20.0 versus 12.2 months (HR: 0.69, 95% CI: 0.56–0.85), PD-L1 >20% 17.7 vs. 13.0 months (HR: 0.77, 95% CI: 0.64–0.92), and PD-L1 >1%, 16.7 versus 12.1 months (HR: 0.81, 95% CI: 0.71–0.93), although the benefit in PFS in the 1-49% population did not reach statistical significance (HR: 0.92, 95% CI: 0.77–1.11), therefore the health authorities only approved the use of pembrolizumab in monotherapy for patients with PD-L1 expression >50% [56]. On the other hand, two negative studies failed to demonstrate a benefit of mono-immunotherapy against CT. Thus, the *CheckMate026* study compared nivolumab at a dose of 3 mg/kg Q2W versus platinum-based CT in patients with stage IV NSCLC with PD-L1 expression >5% in tumor cells and without EGFR- and ALK-activating mutations, but this time the results was negative. The median PFS in the nivolumab group was 4.2 months versus 5.9 months (HR: 1.15; 95% CI: 0.91–1.45; *P*=0.25) in favor of CT, with no differences in OS (14.4 months vs. 13.2 months; HR: 1.02; 95% CI: 0.80–1.30). Again, the toxicity profile was more favorable for nivolumab, with 17.6% Grade 3–4 adverse effects versus 50.6% for those patients who received CT [57]. The grade 3 toxicity recorded in the pembrolizumab studies was also clearly higher for the CT arm (41%) versus pembrolizumab (18%). In the *MYSTIC* study, with randomization to three arms, durvalumab monotherapy also failed to improve RR, PFS, or OS versus CT [58].

#### 6.1.2. Immunotherapy in combination with chemotherapy

Another therapeutic strategy that has become established as a new standard for care practice in certain subgroups of patients is the combination of CT and immunotherapy. The aim is to improve activation of the immune system as a result of chemo-induced immunological effects, namely, a reduction in T lymphocyte activity, increased presentation of tumor antigens, and induction of PD-L1 expression in tumor cells [59].

The first successful results of chemoimmunotherapy were achieved in tumors with non-squamous histology. The *KeyNote 189* study was the first phase III study to demonstrate a greater benefit for the combination of platinum-based CT and pembrolizumab versus CT and placebo in a population of patients with NSCLC EGFR/wild-type ALK and non-squamous histology, including a pre-planned analysis based on PD-L1 expression (negative or positive). This study was positive in terms of its two primary objectives (OS and PFS) and included a pre-planned analysis of the two previous objectives based on PD-L1 expression in the initial design [60]. In a recently published study update, after a 23-month follow-up, the benefit of the combination in terms of OS and PFS was maintained for all subgroups. The median OS was 22.0 (19.5–25.2) months in the combination arm versus 10.7 (8.7–13.6) months in the combination group (HR: 0.56; 95% CI: 0.45–0.70), and the median PFS was 9.0 (8.1–9.9) months in the combination arm versus 4.9 (4.7–5.5) months. In all the subgroups analyzed based on PD-L1 expression, benefit was obtained in PFS and OS: >50%, OS not reached versus 10.1 month, HR: 0.59; 1–49%, OS 21.8 versus 12.1 months, HR: 0.62; <1%, and OS 17.2 versus 10.2 months, HR: 0.52 [61]. The data from final analysis of this study at 31 months post-randomization and having completed the 35-month maximum follow-up have been presented recently. The OS data were still very favorable for the combination in terms of OS (22.0 [19.5–24.5] vs. 10.6 [8.7–13.6] months; HR: 0.56 [95% CI: 0.46–0.69]) and PFS (9.0 [8.1– 10.4] vs. 4.9 [4.7–5.5] months; HR: 0.49 [95% CI: 0.41–0.59]) [62]. The *IMpower 150* study also evaluated the combination of CT and immunotherapy versus CT but adding an antiangiogenic agent (bevacizumab) to the equation. Using a three-arm design and sequential statistical analysis, this study evaluated the role of the quadruple combination carboplatin-paclitaxel-atezolizumab-bevacizumab (ABCP) versus carboplatin-paclitaxel-atezolizumab (ACP) versus carboplatin-paclitaxel-bevacizumab (BCP) [63]. As a differentiating factor in the *KeyNote 189* study, patients with EGFR and ALK mutations were included, although this population represented only a small percentage of cases with respect to the total. The study objectives were PFS and OS in the intention-to-treat population. An initial comparative analysis of the ABCP versus BCP groups was performed, obtaining more favorable data in terms of PFS and OS in the combination with atezolizumab: PFS 8.3 versus 6.8 months; HR: 0.62; *P*<0.001; and OS 19.2 versus 14.7 months; HR: 0.78; *P*=0.02. To optimize the selection of patients most likely to benefit from immunotherapy, a signature was developed based on expression of the effector T cells of certain genes (Teff), namely, PD-L1, CXCL9, and IFN-γ messenger RNA expression. The data were analyzed based on the expression of this Teff signature in the wild-type population, observing a greater benefit in terms of PFS in those patients with high expression of the signature genes versus those with low expression (11.3 vs. 6.8 months; HR: 0.51; *P*<0.001). Another difference in this study was the stratification of patients based on the presence or absence of liver metastases, with even better results in this population. The *IMpower 130* study ran in parallel to *KeyNote 189* and analyzed the value of adding atezolizumab to the combination of platinum-based CT and nab-paclitaxel in patients with non-squamous histology. Addition of this anti-PD-L1 was associated with an increase in OS versus CT alone (18.6 vs. 13.9 months; HR: 0.79, *P*=0.033) and PFS (7 vs. 5.5 months; HR: 0.64, *P*<0.001) [64]. In this same context, the *IMpower132* study, which ran in parallel, analyzed the role of atezolizumab combined with platinum-pemetrexed-based CT versus CT alone in patients with non-squamous histology and found that the combination demonstrated a benefit in terms of PFS (HR: 0.60, *P*<0.001) but not OS (HR: 0.81, *P*=0.08) [65].

In the context of squamous histology, the combination of platinum-based CT and anti-PD-1 versus standard CT has also shown superiority in all the objectives included in the *KeyNote 407* study, the first phase III study to represent a change in the therapeutic standard for a squamous histology. This study involved 559 patients randomized into two arms: One that included standard therapy with carboplatin-paclitaxel or abraxane versus another arm with the same CT and pembrolizumab, maintaining the latter for a maximum of 2 years or 35 cycles. The primary endpoints of the study were PFS and OS. The initial results of the study have been published recently after a median follow-up of 7.8 months. The combination arm showed a higher OS versus standard treatment (15.9 vs. 11.3 months; HR: 0.64, *P*<0.001). With regard to PFS, the data were also favorable for the combination (6.4 vs. 4.8 months; HR: 0.56, *P*<0.001). However, it should be noted that the efficacy data were independent of PD-L1 expression [66]. The *IMpower131* study is equivalent to the previous one but focused on squamous histology. The PFS data available are more favorable for the combination of atezolizumab and carboplatin-nab-paclitaxel compared to CT alone (6.5 vs. 5.6 months; HR: 0.74; 95% *P*<0.03). The OS data are still too limited to conclude if there are any differences in this regard [67]. Consequently, according to the international guidelines that define the treatment of lung cancer, the combination of CT and immunotherapy is considered to be the standard treatment in patients with PDL1 expression <50%, with a demonstrated benefit in phase III studies in terms of survival, RR, and duration of response [68].

#### 6.1.3. Anti-PD1 and anti-CTLA-4 combinations

As an alternative to combinations of CT and immunotherapy, dual inhibition with anti-PD-1 and anti-CTLA4 has been evaluated in various contexts in NSCLC. This strategy aims to provide a complementary enhancement of the immune system at the level of the tumor microenvironment, by blocking the PD-1/PD-L1 pathway, and at the level of peripheral lymphoid organs, by increasing the recruitment of T lymphocytes with antitumor activity through the CTLA-4 pathway [69,70]. Combinations of anti-PD1/PDL1 and anti-CTLA-4 were initially explored in the context of metastatic melanoma, finding long-lasting responses irrespective of PD-L1 expression [71]. To predict the benefit of the combination anti-PD1-1/PD-L1 and CTLA-4 inhibitors, TMB with different cut-off points has been proposed as a predictive marker of benefit for this therapeutic strategy. However, this marker does not correlate with PD-L1 expression; therefore, studies evaluating the value of dual immunotherapy should be stratified based on the TMB and PD-L1 value [72]. The phase III *CheckMate 227* (part 1) study was one of the first phase III studies to evaluate the efficacy and safety of the combination of nivolumab and ipilimumab in the context of stage IV NSCLC versus chemotherapy (carbo-/cisplatin with pemetrexed or gemcitabine) in patients with TMB ≥10 mutations per megabase. The authors reported an increase in RR of 45% versus 26.9% and in PFS (7.2 vs. 5.5 months; HR: 0.58, *P*<0.001) irrespective of PD-L1 levels. However, there were more treatment withdrawals due to toxicity in the dual immunotherapy arm [73]. With regard to the OS in this population, the benefit of the combination with nivolumab and ipilimumab versus CT in the population with PD-L1 >1% or more was 17.1 months versus 14.9 months in favor of the former (*P*=0.007), with this benefit being maintained in the PD-L1-negative population (17.2 vs. 12.2 months) and in the global population irrespective of PD-L1 expression (17.1 vs. 13.9 months) [74]. Other combinations with anti-PD-L1 and anti-CTLA4 have been explored in the *MYSTIC* study, in which patients with advanced NSCLC were randomized to receive durvalumab (anti-PD-L1) versus durvalumab and tremelimumab (anti-CTLA-4) versus platinum-based CT. The primary objectives of the study were OS in the PD-L1-positive population (PD-L1 >25%) in the comparison durvalumab versus CT and PFS in addition to OS in the immuno versus CT combination. In the PD-L1-positive population, the administration of durvalumab was associated with an OS benefit that was close to statistical significance (16.3 vs. 12.9 months; HR: 0.76, *P*=0.04). In the durvalumab and tremelimumab arm, the median PFS was 3.9 versus 5.4 months in favor of CT in the unselected population (HR: 1.05; 99.5% CI: 0.72-1.53; *P*=0.71).However, focusing on the TMB population with >20 mutations/megabase, the median OS was higher in favor of the combination versus CT (21.9 months vs. 10.0 months; HR: 0.49; 95% CI: 0.32–0.74), whereas the toxicity of the immunotherapy combination was higher than for durvalumab but lower than for CT [58]. The *ARTIC* study is a parallel trial in patients previously treated with first- or second-line CT. In this case, two sub-studies were included: Durvalumab versus chemotherapy or durvalumab-tremelimumab versus CT. Sub-study A included the analysis of PD-L1-positive patients (PD-L1 >25%) and showed a benefit in OS of 11.7 months in favor of durvalumab versus 6.8 months for the arm with standard treatment (HR: 0.63), as well as in PFS (3.8 vs. 2.2 months; HR: 0.71). In sub-study B, which compared the duvalumab-tremelimumab combination versus standard treatment, the OS was 11.5 versus 8.7 months (HR: 0.8, *P*=0.1) and PFS was 3.5 months for both groups. Therefore, it was concluded that, in pretreated patients, the combination did not reach statistical significance despite showing an increase in OS [75]. Consequently, although the data for double immunotherapy are favorable in certain subgroups of patients, especially when we select based on TMB, when compared to combinations with chemotherapy and anti-PD-1/PD-L1, the data are more disappointing. It has not been established which TMB cutoff actually predicts the greatest benefit from the combination. Each study has used a predetermined level for each combination, which again represents a stumbling block as regards the real utility of this biomarker. Therefore, as a potential scenario for this therapeutic option, the combination could be considered in those patients not suitable for CT and with a high TMB, or in those tumors not expressing PD-L1. The immune-mediated toxicity in these studies is greater than with mono-immunotherapy, although it is manageable if treated early.

#### 6.1.4. Other combinations

The promising results from the primary analysis of a randomized, double-blind, and placebo-controlled phase II trial evaluating the combination of tiragolumab (an anti-TIGIT monoclonal antibody) with atezolizumab versus placebo and atezolizumab were recently presented at the ASCO2020 conference. In this study, known as *CITYSCAPE*, the addition of tiragolumab to atezolizumab resulted in a significant increase in the RR (37.3% [25–49.6] vs. 20.6% [10.2–30.9]) and median PFS (5.6 [4.2–10.4] vs. 3.9 months [2.7–4.5] [76]).

#### 6.1.5. Real world data

Real world data (RWD) from the front line are scarce. In patients with a PD-L1 expression >50% treated with pembrolizumab, the data available are retrospective and focused on the analysis of efficacy and safety. The data obtained are equivalent to those obtained in phase III studies, although the median OS was more similar to that obtained in the subgroup of PD-L1 patients >50% in *KeyNote 042* than that obtained for patients in the *KeyNote 024* study [77-81]. These differences could be explained, in part, by the percentage of never-smoking patients included in the RWD studies (7–30%), which is higher than the percentage of cases with these characteristics included in the pivotal *KeyNote 024* study. This figure, however, was closer to the cases included in the *KeyNote 042* study, which was around 20% [56]. Another variable that was also more represented in the RWD studies was ECOG 2. First-line real-world studies included more cases with worse ECOG compared to the pivotal studies, a fact that clearly impacted on a worse evolution of these patients and, therefore, a worsening of the OS results compared to the pivotal studies [78-81]. With regard to toxicity, the data from these studies were consistent with the literature. Some authors performed a logistic regression analysis to predict the factors influencing the development of Grade 3 or higher immune-mediated toxicity in the first 3 months after initiation of pembrolizumab. As a result of this analysis, they found that the most strongly associated factor was the presence of an ECOG < 2 [77]. As regards real-world data in patients receiving combinations with CT/anti-PD-L1 versus CT in the different populations explored, no real-world data, except those that include efficiency parameters and a cost-effectiveness analysis, have been published to date. In this sense, there are several studies focused on the squamous and non-squamous population. With regard to the former, the authors found that for the population with PD-L1 <50%, the combination was associated with greater efficiency or cost-effectiveness, as measured in terms of the ICER (incremental cost-effectiveness ratio) versus CT alone. However, these findings were not confirmed in the PD-L1 >50% population, thus suggesting the need for additional follow-up within the pembrolizumab plus CT and pembrolizumab trials to better define cost-effectiveness between these comparators [82]. As regards the non-squamous population, the combination was again associated with greater efficacy and cost-effectiveness, again measured in terms of ICER, versus CT alone in all populations considering PD-L1 expression [82]. Another French study confirmed the profitability of immunotherapy versus CT in the PD-L1 >50% population, in this case adjusting for QUALYs [83]. In summary, RWD in first-line immunotherapy remain limited to date.

### 6.2. ICI in pretreated NSCLC

Until 2014, there were only three drugs approved for the second-line treatment of NSCLC with no driver mutations after progression to previous CT: Docetaxel [84], approved for both the squamous and non-squamous histology; pemetrexed [85], approved only in the non-squamous histology; and erlotinib, approved after failure of at least one previous chemotherapy regimen when other options were not suitable for the patient. However, erlotinib was approved based on the overall population data from the BR.21 study [86]. Efficacy was subsequently evaluated based on the determination of EGFR mutations and a subgroup study was carried out, finding that greater benefit was seen in non-smokers and patients with adenocarcinoma (with a greater probability of presenting an EGFR mutation), and that there was no benefit in terms of OS in those patients lacking an EGFR mutation [87]. New options, such as the combination of docetaxel with antiangiogenics such as nintedanib [88] in patients with adenocarcinoma and ramucirumab, irrespective of histology, subsequently appeared [89]. Immunotherapy, which has led to a transformation in the treatment of patients after progression to a first line and has allowed us to obtain better OS data, with better quality of life and an increasing number of “long-term survivor” patients, appeared in 2015. We will now review the main studies that have evaluated second-line immunotherapy in NSCLC and the data available regarding its use in standard clinical practice.

#### 6.2.1. Phase III studies

The first two immunomodulators to demonstrate success and gain approval in the treatment of NSCLC were two antibodies targeting PD-1, followed by an anti-PD-L1. These three anti-PD-1/PD-L1 antibodies were approved in advanced NSCLC after progression to platinum-based CT as they demonstrated greater efficacy than docetaxel in this context. All of them were compared against docetaxel in randomized phase III studies with a similar design and with the same primary objective of OS, but with some differences that we will discuss below.

The first immunomodulator to be approved in NSCLC was nivolumab, which was evaluated in two separate studies for squamous and non-squamous histologies. In chronological order, the *CheckMate-017* study evaluated the efficacy of nivolumab at a dose of 3 mg/kg Q2W versus docetaxel in 272 patients with advanced squamous cell carcinoma of the lung, demonstrating a clear improvement in OS for the nivolumab arm (9.2 vs. 6 months; HR: 0.59 [95% CI: 0.44-0.79; *P*<0.001]) and also a significant improvement in PFS (3.5 vs. 2.8 months; HR: 0.62 [95% CI: 0.47–0.81; *P*<0.001]) and RR (20% vs. 9%, *P*=0.008). The effect of PD-L1 expression was evaluated according to the prespecified cut-offs of 1%, 5%, and 10%, but was not a prognostic or predictive factor for any of the efficacy endpoints in this study for the squamous histology [90]. The subsequent *CheckMate-057* study evaluated the efficacy of nivolumab with the same regimen versus docetaxel in 582 patients with advanced non-squamous NSCLC, also demonstrating a clear improvement in OS for the nivolumab arm (12.2 vs. 9.4 months; HR: 0.73 [96% CI: 0.59 0.89; *P*=0.002]) and the RR (19% vs. 12%, *P*=0.02). As regards PFS, the nivolumab arm was inferior to docetaxel (2.3 vs. 4.2 months) but superior in terms of the PFS at 1 year (19% vs. 8%; HR: 0.92 [95% CI: 0.77–1.1; *P*=0.39). Likewise, the efficacy was evaluated based on three pre-specified PD-L1 expression cut-off points (≥1%, ≥5%, ≥10%) and benefit was observed at all higher expression levels for each cut-off point, with the magnitude of benefit being greater for higher PD-L1 expression levels [91]. As a result of these studies, nivolumab was granted second-line approval by the FDA in 2015 and by the EMA for squamous and non-squamous histologies in 2015 and 2016, respectively. The other anti-PD-1 that has demonstrated a benefit versus docetaxel in second-line treatment is pembrolizumab. The *KeyNote-010* study was a randomized phase II/III study that compared treatment with pembrolizumab at 2 or 10 mg/kg Q3W versus docetaxel in 1034 patients with advanced NSCLC with positive PD-L1 expression (≥1%) progressing to at least a platinum-based CT regimen. Both doses of pembrolizumab were shown to be superior to docetaxel in terms of OS (10.4 months for pembro 2 mg/kg, 12.7 months for pembro 10 mg/kg, and 8.5 months for docetaxel; HR: 0.71 for pembro 2 mg/kg versus docetaxel [95% CI: 0.58–0.88; *P*=0.0008] and HR: 0.61 for 10 mg/kg vs. docetaxel [95% CI: 0.49–0.75; *P*<0.0001]). There were no significant differences in terms of PFS between the pembrolizumab and docetaxel arms, although both doses of pembrolizumab were also better in terms of RR versus docetaxel (18% for pembro 2 and 10 mg/kg and 9% for docetaxel; *P*=0.0005 for pembro 2 mg/kg versus docetaxel and *P*=0.0002 for 10 mg/kg versus docetaxel]). This study also evaluated the efficacy in the subgroup with high PD-L1 expression (TPS ≥ 50%), which accounted for 28% of the randomized patients, with the OS, PFS, and RR data also being found to be better in this subgroup and showing significant differences in terms of PFS for the two doses of pembrolizumab versus docetaxel (HR: 0.59 [95% CI: 0.44–0.78; *P*=0.001]) [92]. Given these results, pembrolizumab achieved second-line approval for advanced NSCLC with PD-L1 expression ≥1% from the FDA in 2015 (2016 for the EMA). Finally, atezolizumab was also found to be superior to docetaxel in efficacy terms in the randomized phase II POPLAR study and in the phase III randomized OAK study. The OAK study evaluated the efficacy and safety of a fixed IV dose of 1200 mg atezolizumab Q3W versus docetaxel in 1225 patients with advanced NSCLC after progression to a platinum-based CT regimen, irrespective of histology. Atezolizumab was superior in terms of OS (13.8 vs. 9.6 months; HR: 0.73 [95% CI: 0.62–0.87; *P*=0.0003). However, no significant differences were observed in terms of PFS or RR. The improvement in OS was similar in patients with squamous and non-squamous histology. OS was also assessed in the different prespecified PD-L1 expression subgroups (TC1/2/3 or IC1/2/3 [equivalent to expression levels of 1%, 5%, and 50% in tumor cells or immune cells infiltrating the tumor) [93]. Initially, in the first 850 patients, benefit for atezolizumab was observed in all subgroups of PD-L1 expression, including those with negative expression (TC0 or IC0). However, in the update of the study with a median follow-up of 28 months and with the 1225 patients, the OS benefit for the non-expressor subgroup lost statistical significance (HR: 0.77 [95% CI: 0.57–1.03; *P*=0.0766]), although the benefit was maintained in the rest of the PD-L1 expression groups, with the highest magnitude of benefit in the subgroup with high expressors (TC3 or IC3) (HR: 0.45 [95% CI: 0.3–0.68] [94]. Atezolizumab was approved by the FDA in 2016 and by the EMA in 2017.

With regard to toxicity, in general, immunotherapy with anti-PD1/PD-L1 is better tolerated than docetaxel, with fewer adverse effects overall and fewer Grade 3–4 adverse effects, fewer treatment interruptions. In addition, it avoids the hematological toxicity of CT, with less diarrhea, less asthenia, and less nausea and anorexia [90-93,95]. However, it can present immune-mediated toxicity that can affect practically any organ. This is generally mild-moderate, but close monitoring must be put into place because severe immune-related toxicity can appear. Although these serious toxicities are not very frequent, when they appear, the early detection and treatment thereof is essential [96].

One of the greatest successes of immunotherapy has been to increase OS in a previously unfavorable clinical context, namely patients in progression to platinum-based CT or even in highly pretreated patients in progression to several previous lines of CT, thus achieving a “tail” of “long-term survivors” that varies between 17.1 and 23% or 13.4% depending on the time limit used to define “long-term survivors” (at 3 and 5 years). Nivolumab is currently the only agent for which 5-year survival data are available from phase III trials. In a combined analysis of the CheckMate 017 and 057 studies, an OS of 1.1% and 13.4% was observed at 3 and 5 years, respectively (HR: 0.68 [95% CI: 0.59–0.78]) and this long-term benefit is independent of PD-L1 expression. Long-term survivors were also achieved in PD-L1 negative patients, with a 5-year OS of 8% (HR: 0.76 [95% CI: 0.61–0.96]) [97]. Even in highly pretreated patients like those included in the *CheckMate 003* study, a 6-year OS rate of 14.7% was obtained [98]. Three-year OS data are also available for pembrolizumab, with a value of 22.9% (HR: 0.69 [95% CI: 0.6–0.8] in PD-L1 ≥1% and 34.5% (HR: 0.53 [95% CI: 0.42–0.66]) in PD-L1 ≥50%. Similarly, a 3-year OS value of 19% versus 10% in a study update was obtained for atezolizumab in the *POPLAR* study.

In summary, immunotherapy with anti-PD-1/PD-L1 antibodies increases OS versus docetaxel as second-line treatment after progression to at least one platinum-based CT regimen, with better tolerance, better quality of life, and long-term survivor rates not seen to date in this context.

#### 6.2.2. Real world data

Numerous retrospective studies have evaluated the efficacy and safety of second-line and subsequent palliative immunotherapy, especially with nivolumab. Despite the fact that some of them have obtained somewhat inferior results, probably due to the inclusion of a broader population than the clinical trials, these studies tend to confirm the efficacy data from the pivotal studies. They also confirm the safety of use and toxicity in routine clinical practice, with no unexpected toxicities being found. The most interesting information that we can extract from these real-world studies is related to patients with characteristics that are usually excluded from clinical trials, such as the population with ECOG PS2, elderly population, metastases in the CNS or in special populations. Prognostic/predictive factors of efficacy are also evaluated in some of these studies. The studies available with RWD are summarized in the following table. A large number of these studies show that a worse ECOG PS >1 negatively influences the OS, as does the presence of liver metastases, the presence of EGFR or ALK mutations, and the number of metastatic sites [99-115] (Table 4).

**Table 4:** Real world data studies with more than 100 patients evaluating OS with immunotherapy in previously treated advanced NSCLC

Author	Sample size	Treatment	Median OS (months)	Median PFS (months)	Factors influencing OS	Toxicity profile
Garde-Noguera *et al*. [99]	175 2L: 75≥3L: 100	Nivolumab	5.81	2.8	-PS 2 - Time since previous treatment<6 months - Number metastatic locations	Grade 3–4 AE=11.4% Most common AE: fatigue (10.6%), skin toxicity (9.7%)
Areses *et al*. [100]	188	Nivolumab	12.85	4.83	-CNS metastases - ECOG PS	Treatment related AE=78% Grade 3–4 AE=4.8%
Mielgo-Rubio *et al*. [101]	168	Nivolumab 155 Pembrolizumab 13	11.4	5.6	-LIPI index - Antibiotic use	
Rodriguez-Abreu *et al*. [102]	665 2L: 239 3L: 224≥4L: 202	Nivolumab	8.97	3.23		All grade AE=44.5% Grade 3–4 AE=10.4%
Dixmier *et al*. [103]	1837	Nivolumab	11.5			Treatment related AE=31% Grade 3–4 AE=7%
Chouaid *et al*. [104]	10452	Nivolumab	Non-squamous 14.2 Squamous 10.5			
Grossi *et al*. [105]	1588	Nivolumab	11.3	3	-ECOG PS - Liver metastasis	All grade irAE=32% Grade 3–4 irAE=6%
Crinò *et al*. [106]	371	Nivolumab	12.8	4.8	-ECOG PS - Liver metastasis - Bone metastasis	All grade irAE=29% Grade 3–4 irAE=6%
Juergens *et al*. [107]	472	Nivolumab	12	3.5	-ECOG PS - CNS metastasis - EGFR mutations	
Morita *et al*. [108]	901	Nivolumab	14.6	2.1	-ECOG PS - Liver metastasis	All grade irAE=45.8% Grade 3–4 irAE=14%
Khozin *et al*. [109]	1344	Nivolumab and pembrolizumab	8		-EGFR mutations - ALK translocation	
Khozin *et al*. [110]	5257	Nivolumab, pembrolizumab and atezolizumab	9.3	3.2	-EGFR mutations - ALK translocation -Hepatic dysfunction	
Figueiredo *et al*. [111]	219	Nivolumab	13.2	4.9	-ECOG PS	All grade irAE=76.4% Grade 3–4 irAE=18.2%
Karak *et al*. [112]	110	Nivolumab and pembrolizumab	8.1	4		All grade irAE=18%
Ahn *et al*. [113]	155	Nivolumab and pembrolizumab	10.2	3.1	-ECOG PS - EGFR mutations - ALK rearrangement - Liver metastasis	All grade irAE=61.9% Grade 3–4 irAE=5.3%
Nadler *et al*. [114]	1020 2L: 718 3L: 302	Nivolumab and pembrolizumab	2L: 9.7 3L: 11.3			
Weis *et al*. [115]	124	Nivolumab and atezolizumab	Nivolumab: 8.4 Atezolizumab: 6.5	Nivolumab: 2.2 Atezolizumab: 2	-ECOG PS	All grade irAE=70.4/65.1%

### 6.3. ICI in patients with targetable molecular drivers (EGFR, ALK, ROS1, etc.)

In general, ICIs are less effective in NSCLC patients with mutations amenable to target therapy. In the IMMUNOTARGET registry [116], an RR of 26% was observed in KRAS and 24% BRAF, compared to 12% in EGFR and 0% in ALK. More longer responders were observed in the KRAS (25.6%) and MET (23.4%) groups. PFS was associated with PD-L1 expression in KRAS and EGFR and with smoking habit in BRAF and HER2.

#### 6.3.1. Patients with EGFR mutations

In a meta-analysis [117] of the *CheckMate-057* [91], *KeyNote-010* [92], *POPLAR* [118], and *OAK* [93] studies, the use of ICIs was not associated with an increase in OS in previously treated patients with driver mutations. The same was seen with pembrolizumab as first line [119] and in the *KeyNote 001* [120] and *BIRCH* [121] trials, which included previously treated and first-line patients. Hastings *et al*. observed that the RR of patients with exon 19 deletion treated with ICIs was lower than in non-mutated patients (7% vs. 22%, *P*=0.002) but similar to those with mutated exon 21 (*P*=0.42) [122]. A similar situation was observed in IMMUNONOTARGET [116]. It appears that patients with T790M+ obtain less benefit from immunotherapy [123,124], although other studies have found no association [122]. The combination of TKI and immunotherapy has resulted in an increase in toxicity, which has led to the premature closure of some studies, such as TATTON [125] and CAURAL [126], which evaluated the combination of osimertinib and durvalumab. As such, immunotherapy alone, or in combination with the TKIs available in NSCLC, does not appear to be the best treatment strategy in patients with the EGFR mutation. However, promising results were obtained in the *ATLANTIC* study with 12.2% objective responses in the EGFR+/ALK+ population, although the response rate was lower than in the population lacking driver mutations, especially in the subgroup of patients with EGFR mutations included in the *IMpower150* study: The included population presenting mutations in EGFR and translocation in ALK also showed benefit in favor of the combination with carboplatin + paclitaxel + bevacizumab + atezolizumab compared to the data published up until then, which showed little benefit with PD-L1 inhibitors/PD-1 in that type of patient [127]. However, these data were obtained from a sub-analysis with a small number of patients, thus meaning that it is necessary to confirm these findings, as is currently being done in the ABC-lung study (NCT04245085). If confirmed, this combination could become the standard after progression to targeted therapy in these patients.

#### 6.3.2. Patients with ALK rearrangement

The *ATLANTIC* [128] (15 patients), *OAK* [93] (4), and IMMUNOTARGET studies (23 patients) have not observed any response in this group, therefore, despite the small number of patients included, it seems that this subgroup of patients will not benefit from immunotherapy with anti-PD-1/PD-L1 in monotherapy. As in patients with the EGFR mutation, the positive results in terms of OS in the subgroup of patients with EGFR mutations and ALK translocation mean that the carboplatin quadruple + paclitaxel + bevacizumab + atezolizumab is a promising option for patients who can no longer be treated with targeted therapy, as is already being evaluated in the prospective phase II GFPC 06-2018 (NCT04042558) trial.

#### 6.3.3. Patients with other driver mutations

KRAS-mutated adenocarcinomas have elevated TMB [129] and PD-L1 expression [130]. In a meta-analysis, ICIs increased OS versus docetaxel in mutated KRAS (*P*=0.03) [131]. However, the KRAS-TP53 and KRAS-STK11 co-mutation has been associated with lower response [132]. The BRAF mutation is associated with higher PD-L1 expression, low or intermediate TMB and MSI. An RR of 25% and 33% has been observed for V600E and non-V600E, respectively [133]. A study of 147 patients with an alteration in MET exon 14 showed an RR of 17% and PFS of 1.9 months, irrespective of PDL1 and TMB [134]. A recent retrospective series reported an RR of up to 35.7% [135].

## 7. Biomarkers for Immunotherapy

Despite the great progress that immunotherapy has brought about, a biomarker with sufficient sensitivity and specificity to be able to carefully select those patients with the highest probability of response, or to determine those patients who will not benefit from it, has not yet been discovered. Indeed, half of patients with NSCLC will not respond to ICIs. Only PD-L1 expression is currently available in clinical practice, and this is an imperfect biomarker since it has a high positive predictive value but low negative predictive value, thus meaning that a negative result does not exclude a response and some patients with high expression levels will not respond to monotherapy.

### 7.1. PD-L1

The low specificity and sensitivity of this biomarker can be explained by different causes, including determination using different platforms, different antibodies, and different definitions of positive PD-L1 [136]. Furthermore, its expression is heterogeneous and dynamic, with different results between the primary lesion and metastases.

As second-line treatment in the *CheckMate 057* [91], *Keynote 010* [92], and OAK [93] trials, an association was seen between expression thereof, response and survival benefit, with higher expression leading to a higher probability of response. In the *JAVELIN* study, this was only seen in patients with PD-L1 ≥50% [137]. However, it seems that, in squamous histology, the benefit is not as dependent on PD-L1 expression as observed in the *CheckMate 017* study, in which there was no association between PD-L1 expression and benefit in terms of OS and PFS [90].

As first-line treatment in monotherapy, it seems that its value is more decisive. Thus, in the *KeyNote 024* study [26], in patients with PD-L1 ≥50%, and in *KeyNote 042* [56], in PD-L1 ≥1%, pembrolizumab was superior to CT, although an exploratory study with the *KeyNote 042* data found that this benefit was due to the subgroup with PD-L1 ≥50%. In the *CheckMate 026* study, Nivolumab did not increase PFS in patients with PD-L1 ≥5% [57]. Similarly, in the *KeyNote 189* [60], *KeyNote 407* [66], and *IMpower 150* studies [63], the combination of CT and first-line immunotherapy was superior to CT irrespective of PD-L1 expression.

A combination of PD-L1 expression and TMB could improve patient selection. Thus, in *CheckMate 026*, the RR with nivolumab was 2- and 2.2-times higher in patients with higher TMB in the subgroup with PD-L1 expression 1–49% and ≥50%, respectively [138]. Similarly, in *CheckMate 227*, the combination of nivolumab with ipilimumab showed a modest benefit in patients with PD-L1 <1% (HR: 0.74 [0.58–0.94]) but was greater when evaluating only those patients with high TMB (HR: 0.56 [0.35–0.91]) [74].

### 7.2. Tumor Mutational Burden (TMB)

As TMB is also a continuous variable, the different definitions of high/low TMB mean that the results from studies with different cut-off points differ. Smoking patients are known to have higher TMB; therefore, it has been proposed as a clinical surrogate [139]. In the *CheckMate 026* trial, nivolumab was superior to CT in patients with high TMB. There was no relationship between PD-L1 expression and TMB, although patients with both variables elevated had higher PFS [57]. In a retrospective analysis of the MKS-IPACT study [140], and in *CheckMate 012*, TMB was found to be related to efficacy, with higher RR and PFS in patients with high TMB [73].

Similarly, in *CheckMate 227*, a higher PFS (7.2 vs. 5.5 months, *P*<0.001) was observed with ipilimumab-nivolumab versus CT in patients with TMB >10 mut/Mb, irrespective of PD-L1, with no differences in patients with low TMB [73]. However, in the second part of the trial, the benefit of the combination in terms of OS was independent of PD-L1 and TMB [74]. The combination of both biomarkers was unable to identify a subgroup with greater benefit.

A pooled analysis of data from the *KeyNote 010* and *KeyNote 042* trials identified high TMB as being predictive of response [141], although a pooled analysis of the *KeyNote 021*, *KeyNote 189*, and *KeyNote 407* trials did not find the same association [142].

### 7.3. DNA mismatch repair deficiency

Alterations in DNA repair mechanisms through the DNA mismatch repair (MMR) pathway are associated with increased susceptibility to cancer. Tumors with MMR deficiency (dMMR), or with alterations in the polymerases involved in DNA synthesis (POLE, POLD1), have a higher mutational load, which could activate specific T cells and therefore benefit from immunotherapy. High microsatellite instability (MSI-H) is predictive of response to ICIs in some cancers [143], although this phenotype is infrequent in lung cancer (<0.5%) and is generally associated with other markers such as PD- L1 and TMB, therefore, its determination is not justified [144].

### 7.4. Others

A high density of TILs reflects higher immune recognition of tumor cells and an “inflamed microenvironment,” which is associated with higher OS. The predictive value of TILs was also assessed in studies with atezolizumab [93].

As IFNγ can promote cancer cell cytotoxicity, in addition to PD-1/PD-L1, certain genetic signatures combining biomarkers may be associated with a higher probability of response. For example, the effector T cell gene signature (Teff) was studied in the *OAK* trial, and PFS and OS were found to be higher in the group with higher expression [145].

Analytical markers and indices that translate the systemic inflammation status of the host have been described, and a possible association with worse efficacy has been found in those patients with states of greater systemic inflammation. Thus, several retrospective studies have evaluated various indices of systemic inflammation such as the neutrophil/lymphocyte ratio (NLR), the derived neutrophil/lymphocyte ratio (dNLR), the Lung Immune Prognostic Index (LIPI), and the neutrophil/platelets index, among others. Poor PFS and OS outcomes have been reported in those patients with a high NLR [146,147]. Similarly, Mezquita *et al*. developed a prognostic index in lung cancer based on the dNLR and LDH with three prognostic groups (0: good prognosis; 1: intermediate; 2: poor prognosis) [148]. Those patients with LIPI2 had worse efficacy data from immunotherapy, with this group receiving minimal benefit from it [149,150].

A decrease in ctDNA during treatment has been associated with greater benefit [151]. In a pooled analysis of the *OAK* and *POPLAR* trials [152], and in the phase II *B-F1RST* study, benefit was seen with atezolizumab in patients with high blood TMB (bTMB) level [153].

In the future, the search for predictive biomarkers of benefit from immunotherapy will probably be associated with the determination of a combination of several biomarkers that have proven useful by themselves and which can be combined in multivariate models to create immunoscores.

## 8. Other Immunotherapies and Future Perspectives

### 8.1. Vaccinations

Cancer vaccines attempt to strengthen the adaptive immune response with one or more tumor-specific antigens to generate strong and long-lasting immune responses [154]. An increased tumor mutational burden and a higher frequency of tumor neoantigens have been associated with increased responses by T cells [155] and ICIs [139,140,73,149-156]. The vaccines developed in lung cancer comprise peptides, tumor neoantigens, recombinant proteins, dendritic cells, and other adjuvant strategies [157,158]. However, the development of tecemotide [159], belagenpumatucel-L [160], and the MAGE-A3 vaccine has been suspended due to negative results from phase III studies [25]. Results are available for the TG4010 vaccine, which targets the MUC-1 protein and has been shown to be effective in combination with first-line chemotherapy in phase IIB/III, significantly increasing PFS in patients with normal levels of activated triple positive lymphocytes [161]. The negative results can be explained by the fact that they are based on inconclusive phase II studies and the mechanism of action of the vaccines, since their response depends on the immunocompetence of the host, with inhibition of the tumor microenvironment also being important. Moreover, vaccines have the potential to mediate upregulation of PD-L1 in the tumor microenvironment and convert “cold/non-inflamed” tumors into “hot/inflamed” tumors, thus providing a scientific basis for the study of combinations with PD-1/PD-L1 inhibitors [157]. A multicenter phase II trial is currently evaluating TG4010 in combination with nivolumab in pretreated patients with stage IV non-squamous NSCLC (NCT02823990). It is also possible to manufacture vaccines from the patient’s own immune cells, which seems to make more sense in cancer treatment and is typically referred to as personalized immune therapy. However, the identification of predictive biomarkers of response remains essential.

### 8.2. CAR-T cells

The use of genetically modified T cells, particularly those T cells with chimeric antigen receptors (CARs), is becoming the most innovative therapy for the treatment of cancer [162]. These cells are designed to combine the specificity of an antibody, which recognizes surface molecules on tumor cells, with the effector mechanism of a T lymphocyte. These genetically engineered lymphocytes release cytokines in response to antigen-bearing cells, thereby lysing specific target cells [163]. Given the targeted response rates of CD19-targeting T cells in hematologic tumors [164,165], the focus is now on solid tumors. However, this therapy is not exempt from side effects, mainly cytokine lysis syndrome or neurological or digestive effects, which can be serious [166-168]. The target for CAR-T is an antigen present on tumor cells with no or almost no expression in normal cells. The current targets under study in NSCLC are aberrantly overexpressed antigens such as EGFR, MSLN, GPC3, EpCAM, PSCA, MUC1, ROR1, CEA, HER2, FAP, PDL1, CD80/CD86 [162], as well as the combination of these with PD-1 [169], among others. The limitations of this type of therapy lie in the varying expression of these antigens, as the response to CAR-T will be weaker in tumor cells with low antigen expression. Likewise, the study is also based on immunosuppressive substances present in the tumor microenvironment, such as TGFB [170,171], prostaglandin E2, or adenosine [172] as future therapeutic targets for CAR-T.

### 8.3. New therapies and combinations

Vaccines and adoptive cell therapies (CAR-T, TILS, TCR) [173,174] currently remain experimental treatments in solid tumors, with data in tumors such as melanoma being available since the 1980s [175]. We look forward to further progress in the coming years in T-cell engineering, gene editing, as well as cell fabrication and the use of combined strategies to boost the immune system and improve responses with the fewest side effects. A recent example of efficacy improvement in NSCLC involving the combination of two immunomodulators is the CITYSCAPE study, the results of the primary analysis of which have been presented at the ASCO2020 congress. This analysis showed that adding tiragolumab (an anti-TIGIT antibody) to first-line atezolizumab in patients with advanced NSCLC lacking EGFR or ALK mutations significantly increased RR and PFS [76]. As such, the future appears to lie in “personalized immunization”.

## 9. Conclusions

Immunotherapy constitutes a therapeutic strategy that is increasingly present in different contexts of NSCLC. The initial results in pre-treated metastatic patients were the starting point to position it in earlier settings of advanced disease, and even in localized disease, and it is this latter scenario in which numerous studies are underway to evaluate its efficacy. ICIs, anti-PD-1/anti-PD-L1 and anti-CTLA4, were the first immunotherapy agents to be approved for the treatment of NSCLC, although many other alternatives to these inhibitors are being evaluated in numerous clinical trials. Combinations with CT have been found to provide clearly superior results compared to CT in first-line treatment in NSCLC, although the anti-PD-L1/anti-CTLA4 combination is an alternative to consider in certain subgroups of patients. Another strong point of these therapies is the toxicity profile, with better tolerability than CT, although without losing sight of immuno-related phenomena derived from this type of therapy, which can sometimes be serious. The search for biomarkers that allow us to select potential candidates for immunotherapy in a more optimal manner is a priority and has therefore been the subject of intense research over the past few years. We therefore appear to be living in a sweet spot as regards the development of lung cancer treatment, with the rapid development of immunotherapy, which is now also standard treatment in unresectable stage III NSCLC and is also being integrated into earlier stages with promising results from neoadjuvance studies. In addition, we have new strategies for combining immunomodulators that will continue to change the landscape of NSCLC in the coming years.

### Conflicts of Interest

Xabier Mielgo-Rubio declares the following conflicts of interest: Advisory role; Boehringer-Ingelheim, Astra Zeneca, Brystol Myers Squibb. Speakers’ bureau; Roche, Astra Zeneca, Brystol Myers Squibb, MSD, Abbott, Kiowa-Kirin. Research funding; Brystol Myers Squibb. Rest of the authors declare that they have no conflicts of interest.
